# Can China's New Rural Cooperative Medical System Improve Farmers' Subjective Well-Being?

**DOI:** 10.3389/fpubh.2022.848539

**Published:** 2022-02-07

**Authors:** Wenhao Qi, Fang Liu, Tian Zhang, Xiulin Qi

**Affiliations:** ^1^School of Economics and Management, Jilin Agricultural University, Changchun, China; ^2^School of Business, Zhengzhou University, Zhengzhou, China

**Keywords:** subjective well-being, China's new rural cooperative medical system, farmers, Chinese General Social Survey, consumption level

## Abstract

The New Rural Cooperative Medical System (NRCMS) is one of the essential systems for ensuring public health in rural China. This paper investigates the effect of farmers' participation in the NRCMS on their subjective well-being and its mechanisms using data from the Chinese General Social Survey 2017. The results show that farmers' participation in the NRCMS significantly enhances their subjective well-being, and these results remain robust after regression with the instrumental variables method and propensity score matching method. Further analysis of the mechanisms suggests that participation in the NRCMS can enhance farmers' subjective well-being by increasing their consumption levels other than medical consumption. Moreover, medical consumption levels play a negative role in participating in the NRCMS on farmers' subjective well-being, which can be explained as the “masking effect.” The regression results of the subsamples show that the higher a farmer's income is, the less his or her participation in the NRCMS enhances subjective well-being. And the effect of participation in the NRCMS on farmers' subjective well-being is not significant if their health status is too high or too low.

## Introduction

This paper examines the impact of China's New Rural Cooperative Medical System (NRCMS) on farmers' subjective well-being and the mechanisms. In China's basic medical security system, the NRCMS is a vital component. The NRCMS is unique globally, and it was born out of China's rural cooperative medical system during the planned economy period. China's economy was backward and medical resources were scarce at that time. What's more, rural health services were especially not guaranteed under the “urban-rural dual structure.” Against this backdrop, the Chinese government guided rural residents to build a cooperative medical system, the basic logic of which was mutual aid among farmers. However, with the full replacement of the people's commune system by the household contract responsibility system after the 1980s, China's rural cooperative medical care lost its essential organizational support. Then the medical protection for farmers entered a vacuum. This situation did not change until 2002 when the Chinese government proposed the gradual establishment of the NRCMS, which focuses on the coordination of serious diseases. The NRCMS also emphasizes “cooperation” among farmers, but unlike the cooperative medical system in the planned economy, the government began to provide financial support for rural cooperative medical care. The subsidies have been increasing. In 2003, the per capita subsidy for the NRCMS was 10 yuan per year, which has risen to not <550 yuan in 2019, and in 2021 it is expected to be no <580 yuan. At the same time, the coverage of the NRCMS and the scope of reimbursement for related diseases are also gradually expanding. As the most important medical insurance system in rural China, the NRCMS has improved not only farmers' investment, health and quality of life, but also enhanced the utilization of medical services in rural areas (1–4).

Subjective well-being and happiness have been important topics of psychological research and have received substantial attention from social scientists. Easterlin pointed out that the relative increase in real income of residents in developed countries has not increased people's happiness, which is deemed as the famous “Easterlin paradox” (5, 6). Based on this paradox, it is argued that there is a certain threshold of income (7–9), beyond which it is no longer income but other factors that contribute to subjective well-being, including social comparison, income disparity, and family quality of life (10–12). Following this thought, some scholars believed that it is more meaningful to enhance people's happiness than to pursue economic growth for countries that have reached a certain level of economic development (13). Accordingly, the task of social scientists should also shift to the study of happiness (14). China's rapid economic growth over the past four decades has been accompanied by a proliferation of studies on the well-being of the residents (15–18). Studies on Chinese rural residents showed that in addition to income level, health status, educational attainment, housing conditions, medical conditions, and perceptions of social equity could also significantly affect their subjective well-being (19–21). In addition, in rural China, where social security is generally lacking, the implementation of welfare protection programs such as low-income insurance and pensions also improves farmers' subjective well-being (22, 23). However, there is little literature examining the relationship between farmers' subjective well-being and participation in the NRCMS. Most relevant to this paper is the study by Huo and Chen (24). They studied the relationship between participation in the NRCMS and farmers' subjective well-being using two microscopic databases in China and concluded no relationship between them. They pointed out that the reasons were the disadvantages of the NRCMS, such as low reimbursement ratio, narrow reimbursement scope, and cumbersome reimbursement procedures, etc.

With the continuous improvement of the NRCMS and the ongoing changes of factors influencing people's subjective well-being, it is necessary to discuss further the findings and analysis of the relationship between the NRCMS and farmers' well-being in the existing literature. In essence, although there is undoubtedly room for improvement in the operation of the NRCMS, participation in the NRCMS is based on farmers' own choices and represents their “revealed preferences.” This implies that a more meaningful discussion should be on the mechanisms of participation in the NRCMS on the residents' subjective well-being, which has been missing in the theoretical and empirical analysis of the existing literature. Additionally, in identifying the causal mechanisms, the instrumental variables selected in the current literature (e.g., whether family members participate in other medical insurance) have strong endogeneity by the presence of channels that directly affect residents' subjective well-being.

The marginal contributions of this paper are: firstly, the factors influencing farmers' subjective well-being in China have been well-researched, and this paper examines the role of NRCMS, which is a new perspective. Secondly, this paper uses a combination of instrumental variable regression and propensity score matching methods to identify the causal mechanisms between participation in the NRCMS and subjective well-being. In particular, based on some characteristics of the NRCMS in practice, this paper selects household size as an instrumental variable for whether to participate in the NRCMS, which has a strong exogenous nature. Thirdly, this paper carefully analyzes the influence mechanism of participation in the NRCMS on farmers' subjective well-being and reveals the “masking effect” of medical expenditures in promoting farmers' subjective well-being by the NRCMS for the first time.

## Theoretical Analysis and Research Hypothesis

Subjective well-being is a hot research topic in psychology and social science. Generally speaking, psychological researches focus on the psychological mechanisms by which humans obtain well-being, while sociological research focuses more on the external macro and micro factors that affect subjective well-being (25, 26). In recent years, an increasing number of factors have been incorporated into the theoretical and empirical analyses of influences on subjective well-being. Essentially, these factors can be unified in Samuelson's happiness equation, in which one's subjective well-being equals his or her utility divided by desires (27). It implies that the more one gets, or the less one intends to get, the higher one's subjective well-being will be. In terms of what one gets, income is the most fully discussed for subjective well-being (28–30). In addition to this, employment, consumption, and social relationships are also thought to increase one's subjective well-being (31–33). In terms of intentional acquisition, the most profound is the hedonic adaptation theory, which explains why the human acquisition of gains cannot permanently enhance their happiness (34). In addition, people with better self-control also have a higher sense of subjective well-being (35, 36).

Under China's “dual structure,” the income of rural residents is much lower than that of urban residents. In 2020, urban residents' per capita disposable income in China was 43,834 yuan, while rural residents' per capita disposable income was 17,131 yuan, with an income ratio of about 2.56:1 between urban and rural residents.^1^ Low income directly affects subjective well-being, but more importantly, it isn't easy to obtain all necessary goods and services in life, further reducing farmers' subjective well-being. For Chinese farmers, health care is almost the service that most affects their quality of life and well-being. Health care has two basic characteristics compared to other aspects: firstly, it is a much less elastic and rigid need. Individuals can reduce specific consumption in their daily lives to live an affordable life, but few will choose to sacrifice health care coverage. If one becomes ill, the lack of timely treatment can cause great suffering (37). Secondly, some medical expenses are large, especially for rural Chinese residents with low incomes. Given these two characteristics, coupled with the long-standing lack of medical resources in rural China, farmers have long been in the predicament of being afraid of getting sick and seeing a doctor (38). In this context, China's NRCMS provides important and almost unique protection for farmers in medical care. If a farmer enrolled in NRCMS suffers from a disease covered by the claim and receives treatment in accordance with the relevant regulations, he or she will receive different amounts of benefits depending on the circumstances. In addition to the regular subsidies, the NRCMS also focuses on supporting patients with serious diseases. For eight serious diseases such as congenital heart disease in children, the NRCMS subsidizes 70% of the fixed amount of the medical expenditures. And for low-income families and people living on minimum subsistence allowances, the NRCMS has additional subsidies. With the availability of subsidies, sick farmers participating in the NRCMS are more likely to choose to go to the hospital for treatment rather than expecting to heal themselves, which will obviously improve their health level. The improvement of their health status will significantly enhance their subjective well-being. Accordingly, we propose the following hypothesis.

H1: Participation in the NRCMS can improve farmers' subjective well-being.

The level of consumption is an important factor that affects an individual's subjective well-being. Generally speaking, the higher the consumption level an individual enjoys, the happier he or she is (39). Farmers who participate in the NRCMS will have more spending money than non-participants because the latter have to save a large amount of money in case of emergencies. This means that participation in the NRCMS can enhance subjective well-being by increasing consumption other than medical expenses (including shopping, housing, transportation and communication, culture, leisure and entertainment, education and training consumption, etc.).

In the context of the specific circumstances where the NRCMS operates, a more attractive and debatable issue is the impact of participation in NRCMS on medical consumption. The critical point is that even if the NRCMS reimburses part of the medical expenses, it does not automatically mean that farmers' medical costs will decrease (40, 41). On the one hand, farmers participating in the NRCMS may be more willing to choose higher levels of medical services. The design of the NRCMS has taken into account and tries to circumvent this choice of farmers. For example, the reimbursement rate is 60% for village health offices and health clinics, 40% for town health centers, 30% for secondary hospitals, and 20% for tertiary hospitals. Still, participating farmers prefer to go to higher-level hospitals (42). In this case, despite the subsidies from the NRCMS, farmers do not necessarily pay less for treatment than those who don't participate but seek treatment at lower-level hospitals. On the other hand, in reality, doctors often choose different medical treatments and medications depending on whether or not the farmer participates in the NRCMS. The overall cost of treatment will be higher if a farmer is participating the NRCMS but lower otherwise. It also means that even if the subsidized portion of the NRCMS is excluded, the costs for participating farmers are not necessarily smaller than those for non-participants. Moreover, the doctors' optional medical plans do not represent that the patients get higher level of services, and the additional cost would reduce their subjective well-being. Based on the above analysis, we propose the following hypotheses.

H2: Participation in the NRCMS can increase farmers' subjective well-being by increasing overall consumption other than medical care.

H3: Participation in the NRCMS can reduce farmers' subjective well-being by increasing medical consumption.

The impact of participation in the NRCMS on farmers' subjective well-being may vary to some extent depending on farmers' income levels. In China, there is a large income gap between farmers (43, 44). For different farmers with other incomes, participation in the NRCMS has different degrees of significance in improving their subjective well-being. On the one hand, the NRCMS is essentially a financial subsidy for ill farmers, and it can be regarded as an additional income for them. Following the general principle of diminishing marginal returns, the higher income level farmers enjoy, the less increase in subjective well-being such additional subsidies brings. On the other hand, due to the Easterlin paradox, after a certain threshold, income no longer leads to increased subjective well-being but is replaced by other factors such as fairness and respect. Additionally, according to the mechanism by which the NRCMS increases farmers' consumption and thus improves their subjective well-being, the increase in consumption brought about by the NRCMS is more limited for high-income farmers as a proportion of their total consumption. Therefore, the increase in their subjective well-being is also finite. In contrast, the less the farmers earn, the more the additional subsidy may contribute to their consumption behavior of necessities. It may affect their lives more significantly and improve their subjective well-being more markedly.

Differences in farmers' health levels may also lead to differences in the impact of participation in the NRCMS on their subjective well-being. Farmers' health status may vary depending on climate, work, and education level. There is a high probability that farmers in good health do not need medical care because they are less likely to be ill. It means that participation in the NRCMS does not substantially improve their health and thus their subjective well-being. And since healthy farmers have fewer opportunities to receive subsidies from the NRCMS, the impact of participation in the NRCMS on their consumption will be limited, which means that the NRCMS will not enhance their subjective well-being through the mechanism of promoting consumption. Conversely, for farmers with average or poor health, participation in NRCMS can improve their health and increase their consumption, thus ultimately improving their subjective well-being. Based on the above analysis, we propose the following hypotheses.

H4: The higher a farmer's income is, the less subjective well-being he or she gets from participating in the NRCMS.

H5: The healthier a farmer is, the less subjective well-being he or she gets from participating in the NRCMS.

## Research Design

### Data

The data used in this paper are from the China General Social Survey (CGSS) 2017. CGSS is the first national, comprehensive, and continuous large-scale social survey project in China, executed by the China Survey and Data Center of Renmin University of China. The project covers almost all provincial administrative units in mainland China. The survey data of CGSS 2017 was released on October 1, 2020, with a total of 12,582 completed valid samples containing 783 variables, including questions on farmers' participation in the NRCMS and subjective well-being, which were well suited for our study. Because the core task of this paper is to explore whether participation in the NRCMS increases farmers' subjective well-being, only the agricultural households in the sample are retained in this study. After removing observations with missing or abnormal information on key variables, the final sample size is 7,743.

### Variables

#### Dependent Variable

The dependent variable in this paper is subjective well-being (SWB). The CGSS 2017 questionnaire asks respondents about their subjective well-being in detail. In question A36, respondents are asked to answer: “In general, are you happy in your life?” The options included “1. very unhappy; 2. relatively unhappy; 3. not happy or unhappy; 4. relatively happy; 5. very happy.” It may be difficult to accurately distinguish the above five options when reporting their subjective well-being, so we construct a binary variable to reduce the estimation error. The specific assignment rule for SWB is as follows: if the respondent selects “relatively happy” or “very happy,” the value is 1; otherwise, the value is 0. In the subsequent robustness test, we keep the original classification of SWB in the questionnaire for regression with the oprobit model, and assign a value of 1 to SWB when the respondent selects “very happy,” “relatively happy” or “not happy or unhappy.”

#### Independent Variable

The independent variable of this paper is whether residents participate in the NRCMS (Medical_if). In question A61 of the CGSS (2017) questionnaire, “Do you participate in the following social security programs,” respondents are asked about if their participation in the NRCMS. We assign the value of “Medical_if” to 1 for those who chose to participate in the NRCMS and “Medical_if” to 0 for those who did not.

#### Control Variables

There are many factors influencing farmers' subjective well-being. Both age and health status are important factors affecting the subjective well-being of Chinese farmers (45). We introduce age (Age) and health status (Health) as control variables. Due to the traditional background of gender relations, happiness differences in gender exist in East Asian countries (46). We introduce the individual gender variable (Female) as the control variable. In addition, this paper controls for education level (Education), religious affiliation (Religion), minority status (Minority), marital status (Married), fertility status (Children), physical fitness (Health), political status (Party), and individual income (Income) with reference to previous literature (47–50). The assignment methods and statistical descriptions of specific variables are shown in Table 1.

**Table 1:** Variable description and descriptive statistics.

**Variables**	**Definition**	**Mean**	**Standard deviation**	**Minimum**	**Maximum**
**Dependent variable**					
SWB	Yes (1), no (0)	0.750	0.430	0	1
**Independent variable**					
Medical_if	Yes (1), no (0)	0.920	0.270	0	1
**Control variables**					
Age	Age of respondents	50.96	16.22	18	96
Female	Yes (1), no (0)	0.540	0.500	0	1
Education	Non education (0), elementary school (1), middle school (2), college (3), above college (4)	1.440	0.880	0	4
Religion	Yes (1), no (0)	0.890	0.310	0	1
Minority	Yes (1), no (0)	0.0900	0.280	0	1
Married	Yes (1), no (0)	0.770	0.420	0	1
Children	Yes (1), no (0)	0.310	0.460	0	1
Health	Very unhealthy (1), relatively unhealthy (2), average (3), relatively healthy (4), very healthy (5)	3.360	1.150	1	5
Party	Yes (1), no (0)	0.900	0.300	0	1
Income	Log income	8.020	4.330	0	16.12

### Models

#### Probit Model

Since the dependent variable “SWB” is a binary variable, the probit model is chosen to estimate the effect of farmers' participation in the NRCMS on their subjective well-being. The model form is shown in equation (1).


(1) $$\begin{array}{l} SWB_{i}\;=\;\alpha \;+\;\beta {Medical\text{\_}if}_{i}\;+\;\gamma X_{i}\;+\;\varepsilon_{i} \end{array}$$

where *SWB*_*i*_ is a measure of an individual's subjective well-being, *Medical*_*if*_*i*_ is a measure of whether the individual participates in NRCMS or not. *X*_*i*_ are the control variables, and ε_*i*_ is the random disturbance term with the independent identical distribution.

#### Instrumental Variables Regression

The endogeneity problem caused by the two-way causality and omitted variable problem can lead to systematic bias in the probit estimation, so this paper will add a model based on model (1).


(2) $$\begin{array}{l} Medical\text{\_}if_{i}\;=\;1\left[Z_{i}\delta \;+\;\gamma X_{i}\;+\;\nu_{i}\right] \end{array}$$

where 1[·] is the display function, which is taken as 1 when *Z*_*i*_δ + γ*X*_*i*_ + ν_*i*_ > 0, otherwise it is taken as 0. *Z*_*i*_ is an instrumental variable, theoretically highly correlated with rural residents' participation in the NRCMS, but not with their subjective well-being. Both ε_*i*_ and ν_*i*_ are randomly perturbed terms, and *Cov*(ε_*i*_, ν_*i*_) ≠ 0.

#### Propensity Score Matching Method

In this paper, the propensity score matching method (PSM) is used to eliminate the heterogeneity between farmers who participate and do not participate in the NRCMS, thus ensuring the robustness of the findings. Specifically, the sample is assigned a value of 1 if the respondent engages in the NRCMS, and 0 otherwise. The propensity score used for matching is calculated as follows:


(3) $$\begin{array}{l} P\;=\;Pr\left\{DZ_{i}\;=\;1\right\}\;=\;\phi \left\{X_{i}\right\} \end{array}$$

where *P* is the propensity score calculated based on the sample, and *X*_*i*_ are the covariates used to calculate the propensity score. To ensure the robustness of the results, three methods of proximity matching, kernel matching, and radius matching are used for estimation in this paper.

## Empirical Analysis

### Benchmark Regression Results

Columns (1) to (3) of Table 2 report the benchmark regression results. Column (1) is a binary regression result without controlling other variables. The result, which is significant at the 1% level, shows that participation in the NRCMS increases farmers' subjective well-being. Column (2) adds other control variables when column (3) further controls for regional dummy variables for provinces, and the results also show that participation in NRCMS increases farmers' subjective well-being. Both significance levels of the two are still 1%. The regression results fully validate H1.

**Table 2:** Benchmark and IV regression results.

**Variables**	**Probit**			**IV**
	**(1)**	**(2)**	**(3)**	**(4)**
Medical_if	0.2132^***^	0.1816^***^	0.1936^***^	3.0946^***^
	(3.86)	(3.19)	(3.33)	(2.59)
Age		0.0118^***^	0.0112^***^	0.0044
		(8.52)	(7.84)	(0.71)
Female		0.1825^***^	0.1727^***^	0.0841
		(5.44)	(5.05)	(0.94)
Education		0.1701^***^	0.1587^***^	0.0264
		(7.24)	(6.50)	(0.26)
Religion		−0.0024	−0.0197	0.0037
		(−0.05)	(−0.35)	(0.08)
Minority		0.1585^***^	0.2727^***^	0.2250^**^
		(2.63)	(3.59)	(2.01)
Married		0.2788^***^	0.2778^***^	0.0378
		(7.06)	(6.91)	(0.21)
Children		−0.0928^**^	−0.1120^***^	−0.0523
		(−2.19)	(−2.61)	(−0.79)
Health		0.2768^***^	0.2822^***^	0.1833
		(17.67)	(17.14)	(1.52)
Party		−0.3220^***^	−0.3341^***^	−0.1879
		(−5.10)	(−5.21)	(−1.16)
Income		−0.0007	0.0004	−0.0069
		(−0.19)	(0.11)	(−1.50)
Region	No	No	Yes	Yes
Constant	0.4836^***^	−1.2212^***^	−1.0960^***^	−3.0039^***^
	(9.16)	(−8.17)	(−5.73)	(−5.55)
*N*	7,743	7,741	7,741	7,741

*T-statistics are reported in parentheses*;

*** *denotes significance at the 0.01 level*;

** *denotes significance at the 0.05 level*.

The control variables, age, female status, education level, minority status, married status, and physical health are all significantly and positively related to subjective well-being. Having children and being a party member are negatively associated with subjective well-being, probably because family and social burdens are heavier for such individuals. Interestingly, all the regressions show that income does not affect subjective well-being, which constitutes another empirical evidence for the Easterlin paradox. The possible explanation is that the “dual structure” of urban-rural division in Chinese society has not been eliminated, and farmers have more opportunities to be exposed to urban life than before. Hence, the relative income effect generated by the comparison with urban residents dissolves the improvement in subjective well-being brought by the increase in absolute income (51).

### IV Regression Results

There are two sources of endogeneity problems in the benchmark regression: first, omitted variables. Although existing empirical studies continuously explore the factors that influence subjective well-being, it is inherently characterized as challenging to grasp as a personal human feeling. In fact, people can often describe what brings them suffering but have difficulty in clearly stating the reasons for achieving happiness. Coupled with data limitations, empirical evidence with subjective well-being as the dependent variable always has the insurmountable problem of omitted variables. Second, two-way causality. People with higher subjective well-being may be more inclined to participate in the NRCMS. The endogeneity problem brings bias in the estimation, which is corrected by using the instrumental variables approach in this paper.

A qualified instrumental variable needs to satisfy two conditions: first, it is correlated with the endogenous variable; second, there are no other mechanisms to influence the dependent variable other than through the endogenous variable. In this paper, we choose household size, measured by the number of household members, as the instrumental variable, taking into account the specific characteristics of the NRCMS in practice. The reasonableness of the IV is as follows: on the one hand, according to the official regulations, whether to participate in the NRCMS is based on individual wishes. However, many local governments require farmers in the family to participate in the NRCMS as a whole to increase participation (52). It means that the larger a family is, the more it will spend on enrollment. Because of the family's limited income, such a policy of mandatory group enrollment would influence individual enrollment decisions. In addition, there is a “cohort effect” among members of the same household, as it may seem generally unacceptable to others to have only one member of the household enrolled in the NRCMS. From the empirical results, the F-value of the first stage of the two-stage least squares method is 17, indicating no weak instrumental variable problem. On the other hand, there is no other theoretical mechanism for household size to affect farmers' subjective well-being.

Column (4) of Table 2 reports the results of the instrumental variables regressions, indicating that participation in the NRCMS still significantly increases farmers' subjective well-being after dealing with the endogeneity problem. This result reaffirms H1 and demonstrates the robustness of the benchmark regression results.

### PSM Regression Results

We use the propensity score matching method (PSM) to further verify the robustness of the findings. Although the propensity score matching method cannot handle endogeneity problems such as reverse causality and omitted variables, it provides a feasible way to determine causality by constructing homogeneous units. In the case of Chinese farmers, the heterogeneous endowments that influence their decision to participate in the NRCMS are essential barriers to causal judgment. Specifically, we still choose the variables that act as control variables in the above regression to match the original sample. Table 3 shows the balance test between the treatment and control groups after using the nearest neighbor matching method. It can be seen that there are significant differences in age, education, marital status, and income between the control and treatment groups before matching, and these differences are primarily eliminated after matching. All variables in the matched sample except income are no longer statistically significantly different, and the proportion of bias drops to a lower level.

**Table 3:** Balance test.

**Variables**	**Match type**	**Mean**		**Bias (%)**	***T*** **-test**	
		**Treated**	**Control**		***T*-value**	***P*-value**
Age	Before	51.06	49.79	7.5	1.86	0.063
	After	51.033	51.117	−0.5	0.30	0.763
Female	Before	0.53599	0.54072	−0.9	0.23	0.822
	After	0.53637	0.54771	−2.3	1.36	0.175
Education	Before	1.4483	1.3436	11.7	2.81	0.005
	After	1.4456	1.4515	−0.7	0.40	0.687
Religion	Before	0.8914	0.88762	1.2	0.29	0.773
	After	0.89152	0.89139	0.0	0.02	0.981
Minority	Before	0.08685	0.07818	3.2	0.74	0.462
	After	0.08682	0.08213	1.7	1.00	0.315
Married	Before	0.78266	0.67752	23.8	5.99	0.000
	After	0.78261	0.77786	1.1	0.68	0.495
Children	Before	0.30686	0.30293	0.9	0.20	0.839
	After	0.30744	0.29969	1.7	1.01	0.315
Health	Before	3.3596	3.4283	−5.9	−1.42	0.157
	After	3.3602	3.366	−0.5	−0.30	0.766
Party	Before	0.89617	0.90228	−2.0	0.48	0.633
	After	0.89771	0.89547	0.7	0.44	0.662
Income	Before	8.0598	7.5567	11.3	2.77	0.006
	After	8.0573	7.9296	2.9	1.73	0.084

In addition to nearest neighbor matching, kernel matching and radius matching are used to estimate the treatment effects in this paper. Among them, the bandwidth chosen for kernel matching is 0.06, and the radius for radius matching is set to 0.05. The regression results are summarized in Table 4, which shows that participation in the NRCMS has a significant positive effect on farmers' subjective well-being.

**Table 4:** PSM regression results.

	**Neighbor**	**Kernel**	**Radius**
ATT	0.0394^*^	0.0625^***^	0.0650^***^
	(1.85)	(3.13)	(3.26)
N	7,741	7,741	7,741

*T–statistics are reported in parentheses*;

*** *denotes significance at the 0.01 level*;

* *denotes significance at the 0.10 level*.

### Robustness Test Results

To further ensure the robustness of the findings, we conducted the test using a replacement measurement method and a replacement sample. First, we retain the way subjective well-being is measured in the original data and regress it with the oprobit model. The result is shown in column (5) of Table 5. Second, we include individuals who chose “not happy or unhappy” in the category of perceived happiness when constructing the 0–1 variable for subjective well-being and use a probit model to regress. The result is shown in column (6). Third, we restrict the sample strictly to people living in rural or township areas, and the result of the regression using the probit model is shown in column (7). Finally, we construct the variable life satisfaction level (Satisfy) based on question C33 of the questionnaire, “In general, how satisfied are you with your current overall living situation,” replacing the original dependent variable SWB. The specific assignment rule for Satisfy is as follows: if the respondent selects “completely satisfied,” “very satisfied” or “relatively satisfied,” the value is 1; otherwise, the value is 0. We regress the result with the probit model. The result is shown in Column (8). The results indicate that the results above are robust.

**Table 5:** Robustness test results.

**Variables**	**SWB**			**Satisfy**
	**(5)**	**(6)**	**(7)**	**(8)**
Medical_if	0.2942^***^	0.1936^***^	0.3676^***^	0.2161^**^
	(3.34)	(3.33)	(3.15)	(2.09)
Age	0.0201^***^	0.0112^***^	0.0094^***^	0.0121^***^
	(9.70)	(7.84)	(3.18)	(4.98)
Female	0.2598^***^	0.1727^***^	0.1973^***^	0.1227^**^
	(5.52)	(5.05)	(2.91)	(2.03)
Education	0.1791^***^	0.1587^***^	0.1427^***^	0.1542^***^
	(5.21)	(6.50)	(2.94)	(3.64)
Religion	−0.0665	−0.0197	0.0207	−0.0103
	(−0.83)	(−0.35)	(0.19)	(−0.11)
Minority	0.3926^***^	0.2727^***^	0.2969^*^	0.2121
	(3.71)	(3.59)	(1.89)	(1.63)
Married	0.3003^***^	0.2778^***^	0.2339^***^	0.3015^***^
	(4.90)	(6.91)	(2.95)	(4.31)
Children	−0.0728	−0.1120^***^	−0.0909	−0.0705
	(−1.22)	(−2.61)	(−1.02)	(−0.96)
Health	0.4788^***^	0.2822^***^	0.3090^***^	0.2925^***^
	(18.93)	(17.14)	(9.46)	(10.21)
Party	−0.5067^***^	−0.3341^***^	−0.0949	−0.2274^**^
	(−6.64)	(−5.21)	(−0.76)	(−2.16)
Income	−0.0020	0.0004	−0.0078	−0.0042
	(−0.36)	(0.11)	(−1.03)	(−0.60)
Region	Yes	Yes	Yes.	Yes.
Constant		−1.0960^***^	−1.4115^***^	−1.2940^***^
		(−5.73)	(−3.26)	(−3.88)
*N*	7,741	7,741	1,995	2,567

*T-statistics are reported in parentheses*;

*** *denotes significance at the 0.01 level*;

** *denotes significance at the 0.05 level; and*

* *denotes significance at the 0.10 level*.

### Mechanism Analysis

We used a mediated effects test to investigate the mechanism of participation in the NRCMS on farmers' subjective well-being. The equations are as follows.


(4) $$\begin{array}{l} SWB_{i}\;=\;\alpha \;+\;\beta {Medical\text{\_}if}_{i}\;+\;\gamma X_{i}\;+\;\varepsilon_{i} \end{array}$$

(5) $$\begin{array}{l} Intermediary_{i}\;=\;\theta_{i}\;+\;a{Medical\text{\_}if}_{i}\;+\;\eta X_{i}\;+\;\varepsilon_{i} \end{array}$$

(6) $$\begin{array}{l} SWB_{i}\;=\;\theta_{i}\;+\;\delta Medical\_if_{i}\;+\;\rho Intermediary_{i}\\ \;+\mu X_{i}\;+\;\varepsilon_{i} \end{array}$$

where *Intermediary*_*i*_ is the mediating variable. According to the above analysis, the mediating variables *Intermediary*_*i*_ selected in this paper include the total consumption and medical consumption of residents. The former includes consumption other than medical consumption, covering food, clothing, housing consumption, etc.; the latter includes medical costs other than reimbursed expenses by the NRCMS.

The results of the mechanism test are reported in Table 6. Column (9) is the same as column (3) in Table 2, which indicates that participation in the NRCMS increases farmers' subjective well-being. Column (10) shows that participation in the NRCMS increases farmers' total consumption in addition to medical consumption, and column (11) shows that the regression coefficient of whether or not participating in the NRCMS remains significantly positive after including total consumption in the equation. It implies that total consumption plays a partially mediating effect in the enhancement of farmers' subjective well-being by participating in the NPSMS. This result verifies H2.

**Table 6:** Results of mechanism analysis.

**Variables**	**Happiness**	**Total consumption**	**Happiness**	**Medical consumption**	**Medical consumption**
	**(9)**	**(10)**	**(11)**	**(12)**	**(13)**
Medical_if	0.2140^***^	0.5071^***^	0.3175^***^	0.6519^**^	0.3770^***^
	(3.57)	(3.76)	(2.73)	(2.25)	(3.26)
Total consumption			0.0821^***^		
			(3.09)		
Medical consumption					−0.0222^**^
					(−2.05)
Age	0.0117^***^	−0.0238^***^	0.0156^***^	0.0133^**^	0.0136^***^
	(7.92)	(−8.94)	(4.99)	(2.16)	(4.56)
Female	0.1723^***^	0.0588	0.2117^***^	0.1977	0.2204^***^
	(4.89)	(1.03)	(3.17)	(1.37)	(3.31)
Education	0.1642^***^	0.2586^***^	0.1631^***^	0.0335	0.1855^***^
	(6.53)	(6.64)	(3.33)	(0.34)	(3.84)
Religion	−0.0168	−0.2434^**^	−0.0920	−0.3047	−0.1152
	(−0.29)	(−2.56)	(−0.82)	(−1.33)	(−1.03)
Minority	0.2888^***^	0.1075	0.2130	0.0013	0.2295
	(3.69)	(0.83)	(1.43)	(0.00)	(1.54)
Married	0.2831^***^	0.4538^***^	0.2009^**^	0.3509^**^	0.2504^***^
	(6.84)	(6.08)	(2.54)	(2.08)	(3.19)
Children	−0.0906^**^	0.0559	0.0689	0.1297	0.0742
	(−2.05)	(0.84)	(0.78)	(0.72)	(0.84)
Health	0.2793^***^	0.1647^***^	0.3004^***^	−0.5247^***^	0.2989^***^
	(16.43)	(5.47)	(9.16)	(−7.91)	(9.12)
Party	−0.350^***^	−0.1165	−0.2494^**^	0.0499	−0.2624^**^
	(−5.26)	(−1.21)	(−2.01)	(0.18)	(−2.10)
Income	0.0004	0.0214^***^	−0.0086	0.0145	−0.0070
	(0.11)	(2.70)	(−1.08)	(0.84)	(−0.88)
Region	Yes	Yes	Yes.	Yes.	Yes
Constant	−1.173^***^	10.3163^***^	−2.697^***^	7.0264^***^	−1.659^***^
	(−5.96)	(32.89)	(−5.52)	(8.67)	(−4.23)
*N*	7,313	2,092	2,087	2,092	2,087

*T–statistics are reported in parentheses*;

*** *denotes significance at the 0.01 level; and*

** *denotes significance at the 0.05 level*.

A more interesting result comes from the examination of the effect of medical consumption. Column (12) shows that participating in the NPSMS has increased farmers' medical consumption instead. Column (13) shows that after including medical consumption level in the regression equation, participation in the NRCMS on farmers' subjective well-being is still positive. Still, the effect of medical consumption level is negative. It means that the level of medical consumption plays a “masking effect” in the impact of participation in the NRCMS on farmers' subjective well-being, i.e., farmers' involvement in the NRCMS raises the level of medical consumption and ultimately reduces their subjective well-being. The results confirm H3.

### Heterogeneity Analysis

From the regression results of the control variables in the benchmark regression, we find that income does not significantly affect farmers' subjective well-being. To further examine the effect of income heterogeneity, we first divide the sample into three subsamples according to the level of farmers' income and then run the regressions separately. The regression results reported in columns (14–16) in Table 7 show that the economic and statistical significance of participation in the NRCMS on farmers' subjective well-being decreases as income increases. This result validates H4.

**Table 7:** Results of heterogeneity analysis.

**Variables**	**Income**			**Health**		
	**Low**	**Middle**	**High**	**Healthy**	**Average**	**Unhealthy**
	**(14)**	**(15)**	**(16)**	**(17)**	**(18)**	**(19)**
Medical_if	0.2891^***^	0.1930*	0.0701	0.1315	0.2517^***^	0.1444
	(2.86)	(1.90)	(0.59)	(1.16)	(3.09)	(1.01)
Age	0.0120^***^	0.0142^***^	0.0107^***^	0.0157^***^	0.0088^***^	0.0111^**^
	(4.82)	(5.31)	(3.79)	(5.02)	(4.61)	(2.52)
Female	0.2251^***^	0.2009^***^	0.1398^**^	0.0704	0.2260^***^	0.1405
	(3.49)	(3.35)	(2.20)	(1.05)	(4.87)	(1.49)
Education	0.1409^***^	0.1350^***^	0.2263^***^	0.0994^**^	0.1891^***^	0.2852^***^
	(3.20)	(3.14)	(4.86)	(2.15)	(5.75)	(3.85)
Religion	0.0255	0.0406	−0.1831	−0.0239	−0.0323	−0.0448
	(0.27)	(0.40)	(−1.59)	(−0.22)	(−0.42)	(−0.27)
Minority	0.1473	0.5297^***^	0.1744	0.3217^**^	0.3186^***^	0.1379
	(1.20)	(3.94)	(1.10)	(2.28)	(3.04)	(0.64)
Married	0.2026^***^	0.3575^***^	0.3384^***^	0.3128^***^	0.2662^***^	0.5290^***^
	(3.02)	(4.99)	(3.98)	(4.26)	(4.66)	(4.09)
Children	−0.0605	−0.2041^***^	−0.0507	−0.0996	−0.1287^**^	−0.1242
	(−0.70)	(−2.71)	(−0.67)	(−1.01)	(−2.23)	(−1.11)
Health	0.2917^***^	0.3070^***^	0.2249^***^			
	(10.19)	(10.76)	(6.68)			
Party	−0.5689^***^	−0.2930^**^	−0.2475^**^	−0.5177^***^	−0.2776^***^	−0.4477^***^
	(−4.14)	(−2.29)	(−2.57)	(−3.36)	(−3.29)	(−2.91)
Income				0.0073	−0.0006	−0.0045
				(0.99)	(−0.11)	(−0.41)
Region	Yes	Yes	Yes	Yes	Yes	Yes
Constant	−0.8150^**^	−1.3360^***^	−0.9610^***^	−0.6310	−0.1323	−0.1068
	(−1.99)	(−3.17)	(−3.00)	(−1.34)	(−0.57)	(−0.25)
*N*	2,446	2,473	2,354	1,836	4,178	1,272

*T-statistics are reported in parentheses*;

*** *denotes significance at the 0.01 level; and*

** *denotes significance at the 0.05 level*.

The regression results reported in columns (17–19) in Table 7 show that only the sample with average health level significantly improves their subjective well-being by participating in the NRCMS. For both the very healthy and very unhealthy farmers, the effect of participation in the NRCMS on their subjective well-being is not significant. The regression results for the very healthy sample are consistent with H5. The regression results for the unhealthy farmers falsify H5. The possible reasons are: first, the NRCMS does not subsidize all diseases. For serious diseases that can greatly affect well-being, the NRCMS currently covers only 22 of them. It means that if an unhealthy farmer suffers from a disease that the NRCMS does not subsidize, he or she cannot receive the subsidy to improve well-being. Second, even if they can receive the subsidy from the NRCMS, the limited amount of NRCMS claims is inadequate for patients suffering from severe illnesses and chronic diseases requiring long-term treatment. Therefore, it has a minimal effect on their subjective well-being.

## Conclusions

This paper investigates the impact of farmers' participation in NRCMS on their subjective well-being and its mechanisms using data from the CGSS 2017. The results show that farmers' participation in the NRCMS significantly enhances their subjective well-being. These findings remain robust after regression with the instrumental variables method and propensity score matching method. Further analysis of the mechanisms suggests that participation in the NRCMS can enhance farmers' subjective well-being by increasing their overall consumption level in addition to medical consumption. But the level of medical consumption plays a “masking effect” on farmers' subjective well-being, i.e., farmers' participation in the NRCMS increases the level of medical consumption, which eventually reduces farmers' well-being. The regression results of the subsample show that the higher the farmer's income is, the smaller the effect of the participation in the NRCMS on subjective well-being. And the impact of participation in the NRCMS on subjective well-being is not significant for those whose health status is either high or low health.

The following policy recommendations can be drawn from the empirical results. First, the government should pay more attention to farmers' physical and mental health and encourage farmers to participate in the NRCMS by increasing publicity and giving concessions to improve their subjective well-being. Second, the government should pay more attention to helping the needy groups, expanding the scope of reimbursement, increasing the reimbursement amount, and improving the efficiency of the NRCMS. Third, the government should rectify the problem of hospital charges and strictly crackdown on doctors who choose to use different drugs according to whether farmers participate or not so that farmers can really enjoy the preferential treatment of the NRCMS.

Although this paper analyzes in detail that participation in the NRCMS affects farmers' subjective well-being through raising consumption levels, it does not cover the analysis of other related mechanisms. Exploring these mechanisms is a future direction. In addition, China's NRCMS is constantly evolving, and the specific practices vary from place to place. So other future research directions are to understand the evolution of the NRCMS in the macro context of public health protection in China, grasp the differences in the NRCMS in different regions, and identify the resulting differences in performance.

## Data Availability Statement

Publicly available datasets were analyzed in this study. This data can be found here: http://cnsda.ruc.edu.cn/index.php?r=projects/view&id=94525591.

## Author Contributions

WQ and XQ contributed to the conception of the manuscript and wrote the manuscript. TZ and FL collected the materials and data. WQ, XQ, TZ, and FL contributed to the analysis or interpretation of data. All authors contributed to the article and approved the submitted version.

## Funding

This research was partly supported by the National Social Science Fund of China (21CJL018).

## Conflict of Interest

The authors declare that the research was conducted in the absence of any commercial or financial relationships that could be construed as a potential conflict of interest.

## Publisher's Note

All claims expressed in this article are solely those of the authors and do not necessarily represent those of their affiliated organizations, or those of the publisher, the editors and the reviewers. Any product that may be evaluated in this article, or claim that may be made by its manufacturer, is not guaranteed or endorsed by the publisher.

## Footnotes

^1^Data from the National Bureau of Statistics.
